# Color Stability of Microhybrid Composite Resins Depending on the Immersion Medium

**DOI:** 10.1055/s-0044-1791762

**Published:** 2024-11-21

**Authors:** Sabra Jaâfoura, Amira Kikly, Meriem Fejjeri, Sarra Nasri, Mariem Brini, Dorra Kammoun

**Affiliations:** 1 Department of Dental Biomaterials, Faculty of Dental Medicine, University of Monastir, Monastir, Tunisia; 2 Laboratory of Dento-Facial, Clinical and Biological Approach (ABCDF) LR12ES10, University of Monastir, Avicenna Avenue, Monastir, Tunisia; 3 Laboratory of Research in Oral Health and Oral-Facial Rehabilitation (LR12ES11), Monastir, Tunisia; 4 Department of Conservative Odontology, Faculty of Dental Medicine, University of Monastir, Monastir, Tunisia; 5 Department of Restorative Dentistry and Endodontics, Faculty of Dental Medicine, University of Monastir, Monastir, Tunisia; 6 Research Laboratory of Occlusodontics and Ceramic Prostheses LR16ES15, Department of Fixed Prosthodontics, Faculty of Dental Medicine of Monastir, University of Monastir, Monastir, Tunisia

**Keywords:** composite resins, color, immersion, spectrophotometer, esthetics, dental

## Abstract

**Objective** The aim of this study was to assess the color stability of two microhybrid composite resins after immersion in different coloring solutions for 4 weeks.

**Materials and Methods** Sixty disc-shaped samples (2 mm in thickness and 10 mm in diameter) were prepared according to ISO standard 4049. Two composite resins were used: Filtek Z350 XT (3M ESPE) and Harvard Restore (Harvard Dental International GmbH). After initial color measurements, five samples of each resin were immersed in artificial saliva, Turkish coffee, lemonade, black tea, Coca-Cola 0%, and green tea for 4 weeks. The spectrophotometric measurements were carried out after 24 hours of immersion in the various solutions and then weekly, using a VITA Easyshade Advance 4.0 spectrophotometer (CIE L*a*b* system). Statistical analysis was carried out with SPSS 25.0 software.

**Results** The two composite resins tested revealed discoloration after immersion in all the drinks at a variable immersion period showing different color behaviors. The one-way analysis of variance showed a significant difference in the values of brightness (L), in chromaticity from green to red (a), in chromaticity from blue to yellow (b), and in the color (ΔE) of the two materials at different time intervals. The greatest color change in all the groups was caused by coffee followed by lemonade and black tea followed by green tea, Coca-Cola 0%, and artificial saliva.

**Clinical Significance** The importance of color stability of dental restorations is crucial for dental professionals and patients. Indeed, the quality of a restoration is considered from both a functional and esthetic points of view. The information obtained from this study should prove useful for clinicians to make informed decisions in selecting the best materials for their patients' esthetic restorations.

**Conclusion** The Harvard Restore showed a better colorimetric behavior compared with the Filtek Z350. Coffee, black tea, and green tea had the most marked effects on the discoloration of composites, especially on Filtek Z350. Coca-Cola 0% showed a similar behavior to artificial saliva.

## Introduction

The esthetic appearance of composite resins is a major factor in their use in restorative dentistry. To be considered clinically acceptable, these materials must not only provide an initial color match but also maintain the long-term esthetic appearance of the restored tooth. 1 This color stability is defined as the ability of any dental material to retain its original color. Therefore, color behavior can be considered an important criterion for the use of restorative material in an esthetically critical area. 2 3 4

The oral cavity is a dynamic environment. With the continuous presence of microflora, saliva, and frequent consumption of colored foods (chromatogens), the color stability of a material can be compromised. This property is often neglected when selecting a restorative material over other physical and mechanical properties. Staining or discoloration is one of the primary reasons for replacement of composite restorations. Discoloration of the composite resin surface is a complex phenomenon that may involve several mechanisms such as discoloration by adsorption/absorption of dyes from exogenous sources such as nicotine and tea, coffee, and other beverages. 5

Despite advances in their manufacture, the color stability of composite resins over time remains a major problem and leads to patient dissatisfaction and the need to replace these restorations, which involves spending more time and money. 6

A spectrophotometer is a scientific equipment used for color matching and measurement. It gives information about the reflectance curve as a function of wavelengths in the entire visible range, and thus numerically specifies the perceived color of an object. 7 8

Spectrophotometry has been reported as a reliable technique for performing quantitative color assessment in dental materials studies. To evaluate color differences, the American Dental Association has recommended the use of the CIE L∗ a∗ b∗ (Commission Internationale de I 'Eclairage L∗ a∗ b∗) measurement system, which is well suited for determining minor color differences and has been widely used in dentistry. 9 10

We propose, through this *in vitro* study to explore the colorimetric behavior of Filtek Z350 XT (3M ESPE) and Harvard Restore (Harvard Dental International GmbH) which are two microhybrid composite resins used for definitive coronal fillings, and after immersion in different solutions.

The immersion solutions used were artificial saliva, Coca-Cola 0%, black tea, green tea, Turkish coffee, and lemonade.

We started with three null hypotheses, namely:

1. The duration of immersion does not influence the color stability of the two composite resins tested.
2. The two tested materials have a similar colorimetric behavior.
3. The color is not influenced by the type of immersion solution.

## Materials and Methods

### Materials Studied

Two commercially available resin composite materials were used ( Table 1 ).

**Table 1:** The composition of the two tested composite resins

Composite resin	Manufacturer	Composition	Lot number	Shade
Filtek Z350 XT	3 M ESPE, Minnesota, United States	Bis-GMA, UDMA, Bis-EMA, TEGDMA; the fillers are a combination of nonagglomerated/nonaggregated 20-nm silica filler, 4–11 nm nonagglomerated/nonaggregated zirconia filler, and zirconia/silica aggregates cluster filler (consisting of 20-nm silica and zirconia particles of 4–11 nm)	N782805	Dentine A2
Harvard Restore	Harvard Dental International GmbH, Germany	Bis-GMA, inorganic charged particles with a size of 0.05–1.5 μm. The total filler content is 81% (by weight) and 65% (by volume)	91603932	A2

Abbreviations: Bis-EMA, ethoxylated bisphenol A dimethacrylate; Bis-GMA, bisphenol A glycidyl dimethacrylate; TEGDMA, triethylene glycol dimethacrylate; UDMA, urethane dimethacrylate.

### Sample Preparation

For each material, 30 disc-shaped samples (2 mm thick and 10 mm in diameter according to ISO standard 4049) were prepared with a mold ( Table 2 ).

**Table 2:** Distribution of samples according to material

Material	Shade	Lot number	Number of samples
Filtek Z350 XT	Dentine A2	N782805	30
Harvard Restore	A2	91603932	30

**Fig. 1:**
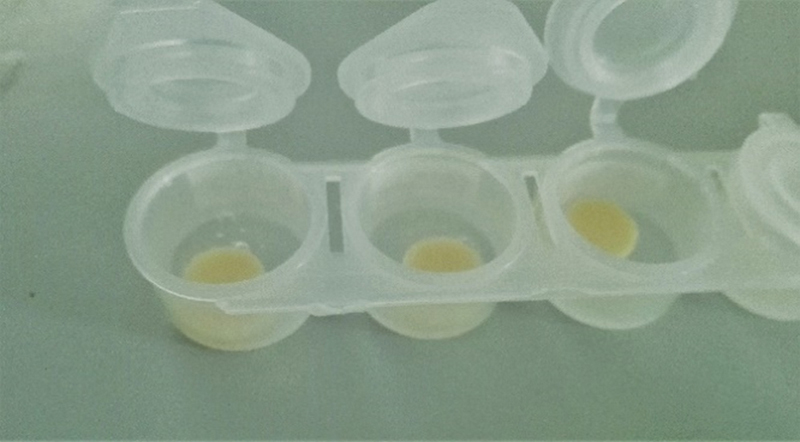
Photograph of prepared specimens.

The resin was condensed with a spatula, and the mold was compressed between two glass plates to remove the excess material and obtain a flat, smooth surface. Each disc was light-cured through the glass plate for 40 seconds for each side (LED photopolymerization lamp: ST-10B, Ultradent, 420–480 nm, >1,000 mW/cm ^2^ ). To ensure adequate curing, the specimens were cured for an additional 40 seconds after the removal of the glass plates before proceeding to polish which was performed by the same operator using a handpiece (NSK Ultimate XL Micromotor) and a polishing wheel ( Fig. 1 ). All samples were stored at 37°C in distilled water for 24 hours for rehydration.

### Immersion of Samples in Different Solutions

After 24 hours of storage in distilled water, for each resin, samples were randomly divided into six subgroups ( *n* = 5).

- Group A: immersion in artificial saliva as a control group.
- Group B: immersion in Turkish coffee (to prepare this solution, we used two teaspoons (Ben Yedder pure Turkish coffee) flush in 100 mL of water.
- Group C: immersion in lemonade.
- Group D: immersion in black tea (to prepare this solution, we used a bag of black tea infusion 10 seconds SPIPA of 1.75 g, reference: PF31 in 100 mL of water).
- Group E: Coca-Cola 0% (pH: 2.8).
- Group F: Green tea (to prepare this solution, we used a bag of green tea with mint infusion 10 seconds SPIPA of 1.75 g, reference: PF44).

**Fig. 2:**
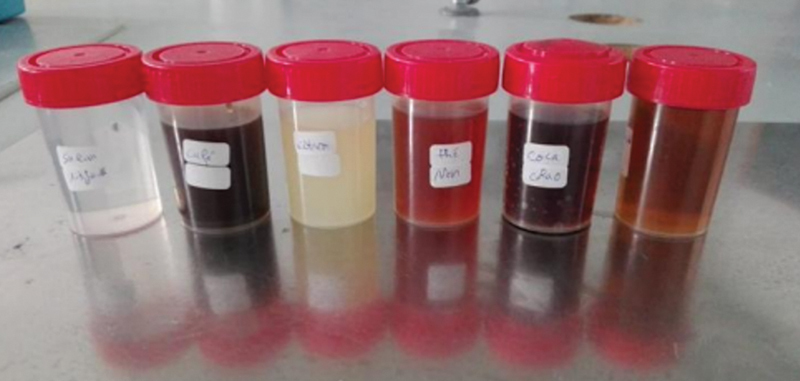
The different immersion solutions used.

The pH of each solution ( Table 3 ) was measured using a pH meter (CyberScan pH 510, Eutech Instruments). The different solutions used are illustrated in Fig. 2 .

**Table 3:** The pH of the different solutions used in the study

Solution	pH	Manufacturer
Artificial saliva	6.7	Chemistry Laboratory of the Faculty of Dental Medicine
Coffee	6.07	Ben Yedder Coffee, 7, Avenue Dag Hammarskjoeld 1001, Tunis, Tunisia
Lemonade	3.31	Homemade
Black tea	6.21	SPIPA, 21, Street of Energy-La Charguia, Carthage, 2035 Tunis, Tunisia
Coca-Cola 0%	2.84	Company of Drinks of Tunisia, Earth Avenue, North Urban Center 1082, Tunis, Tunisia
Green tea	6.94	SPIPA, 21, Energy Street, La Charguia, Carthage, 2035 Tunis, Tunisia

**Fig. 3:**
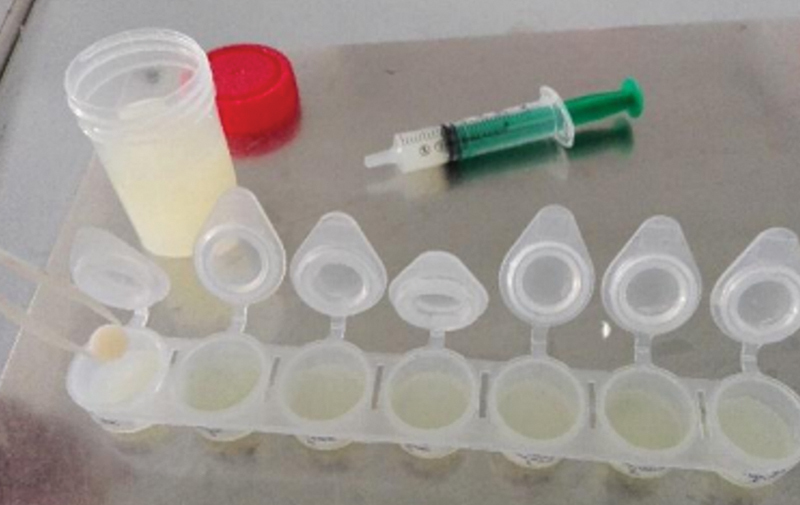
Immersion of samples in lemonade.

For the immersion of the samples in the solutions, we proceeded as follows:

- Using a single-use syringe, 2 mL of the relevant solution was dropped into the well, and then using the prescreen, we placed one sample into each well ( Fig. 3 ).
- Labels on the containing cups bearing the nature of the solution used; the composite resin used; and the number of samples were performed.
- Finally, all the samples were kept in the oven at 37°, and during the immersion period, the solutions were renewed every other day to avoid contamination by bacteria or yeasts.

### Spectrophotometric Measurements

Spectrophotometric measurements were performed using a VITA Easyshade Advance spectrophotometer 4.0 (VITA Zahnfabrik H.Rauter GmbH & Co., Bad Säckingen, Germany) at T0 (before immersion in the different solutions), then after 24 hours of immersion, 1 week, 2 weeks, 3 weeks, and 1 month of immersion in the staining solutions (Arocha et al, 2013). 11

Before each measurement session, the spectrophotometer was calibrated according to the manufacturer's recommendations. The samples were washed with distilled water for 1 minute and dried with absorbent paper. The color was evaluated using the CIE L* a* b* (International Commission on Illumination L* a* b*) measurement system.

CIE-Lab is expressed by the coordinate L*, representing the brightness of the color, ranging from zero (black) to 100 (white), and the coordinates a* and b*, representing the chromaticity of the color, with axes varying from green to red and from blue to yellow, respectively. Positive values of a* indicate a shift to red, while negative values indicate a shift to green. Similarly, positive values of b* indicate the yellow color range, while negative values indicate the blue color range. 12

Color measurements were made at the center of the disks and repeated three times. Color change was calculated from the average ΔL*, Δa*, and Δb* values for each specimen using the following formula: ΔE* = ([ΔL*] ^2^ + [Δa*] ^2^ + [Δb*] ^2^ )1/2.

The entire procedure was carried out by a single operator. To avoid bias, a second investigator evaluated the tested samples randomly.

Since interexaminer variability was not significant, the results of the previous assessment were considered, and the values were totaled and subjected to statistical analysis.

### Statistical Analysis

Statistical analysis was performed using SPSS Statistics 25.0 software. Data showed normal distribution using Kolmogorov–Smirnov's test. Continuous data were described using mean and standard deviation. Intergroup comparison was performed using a two-way analysis of variance (ANOVA) followed by Tukey's post hoc test. A *p* -value of less than or equal to 0.05 was considered statistically significant with a confidence level of 95% and a power of 80%, and all tests were two-tailed.

## Results

### Changes in CIE Lab Parameters

#### Comparison of Color Coordinates Based on Immersion Solution for the Filtek Z350 XT

Descriptive data relating to the variation of the color coordinates ΔL, Δa, and Δb depending on the immersion solution are represented in Tables 4 5 to 6 .

**Table 4:** Descriptive data of ΔL for the Filtek Z350 XT

Immersion medium		ΔL1 Filtek	ΔL2 Filtek	ΔL3 Filtek	ΔL4 Filtek	ΔL5 Filtek
Artificial saliva	Median	1.60	−0.20	1.00	1.10	−0.70
	Minimum	−0.40	−2.80	−1.20	−0.90	−4.00
	Maximum	5.00	2.60	3.00	2.80	2.50
Coffee	Median	9.50	19.00	17.40	22.00	22.00
	Minimum	6.30	13.30	11.40	17.10	13.50
	Maximum	12.00	26.60	23.20	26.80	26.30
Lemonade	Median	0.90	3.20	3.40	4.00	5.10
	Minimum	−0.10	0.50	2.00	2.90	3.30
	Maximum	1.40	4.30	6.20	5.40	9.20
Black tea	Median	4.10	8.20	10.20	12.80	12.80
	Minimum	2.50	6.20	8.80	8.50	5.50
	Maximum	6.00	9.00	11.90	15.60	15.60
Coca-Cola 0%	Median	1.30	2.20	0.70	2.80	1.30
	Minimum	0.00	0.40	−0.70	1.40	−0.20
	Maximum	1.50	2.90	1.70	3.60	2.60
Green tea	Median	2.80	5.30	6.00	8.80	6.00
	Minimum	1.10	2.50	4.90	5.70	5.10
	Maximum	6.50	7.60	7.60	10.30	10.30

**Table 5:** Descriptive data of Δa for the Filtek Z350 XT

Immersion medium		Δa1 Filtek	Δa2 Filtek	Δa3 Filtek	Δa4 Filtek	Δa5 Filtek
Artificial saliva	Median	0.30	1.00	0.80	0.70	0.80
	Minimum	0.00	0.50	0.60	0.40	0.20
	Maximum	0.30	1.30	1.00	1.20	1.20
Coffee	Median	−5.70	−8.90	−8.60	−10.70	−10.50
	Minimum	−7.90	−9.40	−9.80	−11.80	−11.10
	Maximum	−3.00	−5.20	−6.90	−9.70	−9.20
Lemonade	Median	1.90	2.20	2.70	2.40	1.30
	Minimum	0.00	1.90	2.30	1.50	1.00
	Maximum	2.10	2.80	3.10	3.30	2.50
Black tea	Median	−1.00	0.90	1.80	1.80	1.80
	Minimum	−1.10	0.30	−0.10	0.40	0.40
	Maximum	1.20	1.80	2.10	2.20	2.20
Coca-Cola zero%	Median	−0.10	−0.50	0.00	0.90	−1.20
	Minimum	−0.50	−0.70	−0.70	−1.40	−1.30
	Maximum	0.50	0.30	0.20	0.10	−0.90
Green tea	Median	−1.20	−1.60	−1.40	−2.20	−1.60
	Minimum	−3.50	−2.50	−1.60	−4.00	−4.00
	Maximum	−0.60	−1.20	−1.10	−0.50	−0.50

**Table 6:** Descriptive data of Δb for the Filtek Z350 XT

Immersion medium		Δb1 Filtek	Δb2 Filtek	Δb3 Filtek	Δb4 Filtek	Δb5 Filtek
Artificial saliva	Median	1.00	1.00	0.50	1.00	0.80
	Minimum	−1.00	−2.20	−1.40	−0.40	−2.60
	Maximum	2.00	2.80	1.90	1.90	4.90
Coffee	Median	−15.40	−15.10	−15.00	−16.10	−15.90
	Minimum	−16.90	−16.00	−16.80	−17.80	−17.80
	Maximum	−11.10	−10.30	−13.40	−14.20	−14.00
Lemonade	Median	−9.20	−21.00	−26.90	−27.50	−35.50
	Minimum	−9.60	−25.70	−29.30	−30.00	−38.20
	Maximum	−5.90	−17.50	−22.20	−24.60	−32.90
Black tea	Median	−7.30	−13.20	−23.30	−26.50	−26.50
	Minimum	−8.20	−17.00	−25.80	−29.20	−29.20
	Maximum	−2.80	−12.10	−20.60	−20.40	−19.30
Coca-Cola zero%	Median	−0.50	−1.90	−0.80	−2.90	−1.00
	Minimum	−2.50	−2.50	−3.50	−3.60	−4.20
	Maximum	1.00	−0.40	−0.70	1.10	−0.50
Green tea	Median	−6.30	−10.20	−12.60	−7.50	−7.50
	Minimum	−7.40	−13.40	−13.80	−12.90	−13.20
	Maximum	−2.30	−7.20	−7.30	−5.30	−5.30

The numbers assigned for each parameter are coded as follows: 1: T0-24 hours, 2: 24 hours to 1 week, 3: 1 week to 2 weeks, 4: 2 weeks to 3 weeks, and 5: 3 weeks to 4 weeks.

For the Filtek Z350 XT, the ANOVA test showed a highly significant difference ( *p* = 0.000) in Δa values at different time intervals (i.e., Δa1, Δa2, Δa3, Δa4, Δa5) between the combined groups.

Tukey's test was applied to compare the variables two by two. At 24 hours, the coffee showed a significant variation ( *p* = 0.000) in chromaticity from green to red compared with artificial saliva and the other solutions.

After 1 week of immersion, green tea significantly modified the chromaticity from green to red compared with the other solutions except Coca-Cola 0% ( *p* = 0.164). Black tea, lemonade, and Coca-Cola 0% showed similar behavior to artificial saliva. At 2 weeks, chromaticity continued to vary significantly for green tea and coffee. At 3 weeks of immersion, lemonade significantly influenced chromaticity. Black tea and Coca-Cola 0% have chromaticity values similar to artificial saliva. After 1 month, the chromaticity from green to red varied significantly for Coca-Cola 0%, coffee, and green tea. Coca-Cola 0% had a similar behavior to green tea.

The ANOVA test showed a highly significant difference ( *p* = 0.000) in Δb values at different time intervals (i.e., Δb1, Δb2, Δb3, Δb4, Δb5) between the combined groups.

The comparison of the different variables by the Tukey's test showed a significant variation in the chromaticity values from blue to yellow for coffee, lemonade, black tea, and green tea compared with artificial saliva after 24 hours of immersion ( *p* = 0.000).

Coca-Cola 0% exhibited blue to yellow chromaticity similar to artificial saliva at different time intervals up to 1 month. Coffee, black tea, and green tea had similar blue to yellow chromaticity values ( *p* < 0.000 with artificial saliva) for up to 1 week. At 2 weeks, the chromaticity values from blue to yellow varied significantly between coffee and black tea ( *p* = 0.000). After 1 month, the variation was significant between the different solutions tested ( *p* = 0.000) except between artificial saliva and Coca-Cola 0% ( *p* = 0.695).

The ANOVA test showed a highly significant difference ( *p* = 0.000) in ΔL values at different time intervals between the combined groups. The analysis of the variables two by two by the Tukey's test showed luminosity values similar to those of artificial saliva for lemonade, black tea, green tea, and Coca-Cola 0%. Coffee caused a significant variation ( *p* = 0.000) in brightness with artificial saliva after 24 hours. At 1 month, luminosity varied significantly between artificial saliva and black tea, green tea, and coffee ( *p* = 0.000, 0.008, and 0.000, respectively).

#### Comparison of Color Coordinates Based on Immersion Solution for Harvard Restore

Descriptive data relating to the variation of the color coordinates ΔL, Δa, and Δb depending on the immersion solution are represented in Tables 7 8 to 9 .

**Table 7:** Descriptive data of ΔL for the Harvard Restore

Immersion medium		ΔL1 Harvard	ΔL2 Harvard	ΔL3 Harvard	ΔL4 Harvard	ΔL5 Harvard
Artificial saliva	Median	1.30	0.40	1.20	0.80	−0.10
	Minimum	0.80	0.10	0.90	0.70	−1.50
	Maximum	1.60	1.10	2.60	1.40	0.60
Coffee	Median	9.50	19.00	17.40	22.00	22.00
	Minimum	6.30	13.30	11.40	17.10	13.50
	Maximum	12.00	26.60	23.20	26.80	26.30
Lemonade	Median	1.40	6.30	6.10	10.00	9.30
	Minimum	0.30	4.80	5.40	7.70	4.60
	Maximum	4.70	7.60	7.70	14.00	13.40
Black tea	Median	0.90	3.20	3.40	4.00	5.10
	Minimum	−0.10	0.50	2.00	2.90	3.30
	Maximum	1.40	4.30	6.20	5.40	9.20
Coca-Cola 0%	Median	0.90	1.70	1.60	1.30	0.80
	Minimum	0.20	−0.40	0.30	−0.60	−0.90
	Maximum	3.90	9.30	4.20	4.10	2.60
Green tea	Median	2.30	7.90	10.20	11.20	11.20
	Minimum	0.80	6.90	8.80	9.60	8.70
	Maximum	5.00	8.50	11.90	13.20	13.20

**Table 8:** Descriptive data of Δa for the Harvard Restore

Immersion medium		Δa1 Harvard	Δa2 Harvard	Δa3 Harvard	Δa4 Harvard	Δa5 Harvard
Artificial saliva	Median	−0.20	0.20	0.60	0.10	0.00
	Minimum	−0.50	−0.30	0.20	−0.30	−0.50
	Maximum	0.20	0.80	1.30	1.10	0.50
Coffee	Median	−5.70	−8.90	−8.60	−10.70	−10.50
	Minimum	−7.90	−9.40	−9.80	−11.80	−11.10
	Maximum	−3.00	−5.20	−6.90	−9.70	−9.20
Lemonade	Median	−0.90	0.00	−0.20	−1.80	−2.80
	Minimum	−1.30	−0.50	−0.40	−3.10	−3.70
	Maximum	−0.40	0.10	0.00	−1.40	−1.80
Black tea	Median	1.90	2.20	2.70	2.40	1.30
	Minimum	0.00	1.90	2.30	1.50	1.00
	Maximum	2.10	2.80	3.10	3.30	2.50
Coca-Cola 0%	Median	0.00	0.60	1.20	0.90	0.90
	Minimum	−0.30	−0.10	0.50	0.10	0.10
	Maximum	0.30	1.20	1.80	1.80	1.60
Green tea	Median	−0.40	−0.50	−2.30	−2.80	−4.00
	Minimum	−1.60	−1.30	−3.20	−5.50	−5.60
	Maximum	1.00	0.90	−2.00	−2.10	−2.30

**Table 9:** Descriptive data of Δb for the Harvard Restore

Immersion medium		Δb1 Harvard	Δb2 Harvard	Δb3 Harvard	Δb4 Harvard	Δb5 Harvard
Artificial saliva	Median	−1.10	−1.30	−1.20	−1.90	−1.70
	Minimum	−2.70	−3.10	−1.90	−4.10	−3.10
	Maximum	1.10	1.20	1.20	0.30	0.00
Coffee	Median	−15.40	−15.10	−15.00	−16.10	−15.90
	Minimum	−16.90	−16.00	−16.80	−17.80	−17.80
	Maximum	−11.10	−10.30	−13.40	−14.20	−14.00
Lemonade	Median	−8.70	−11.50	−13.40	−18.40	−18.80
	Minimum	−10.90	−13.70	−15.30	−20.90	−21.50
	Maximum	−4.90	−9.90	−10.80	−17.50	−17.90
Black tea	Median	−9.20	−21.00	−26.90	−27.50	−35.50
	Minimum	−9.60	−25.70	−29.30	−30.00	−38.20
	Maximum	−5.90	−17.50	−22.20	−24.60	−32.90
Coca-Cola 0%	Median	−2.60	−5.00	−5.00	−5.20	−7.50
	Minimum	−3.70	−8.20	−7.80	−8.70	−8.60
	Maximum	−1.70	−1.30	−1.80	−2.40	−4.20
Green tea	Median	−4.80	−12.20	−19.80	−26.00	−28.60
	Minimum	−7.20	−19.30	−21.50	−32.30	−32.30
	Maximum	0.00	−8.50	−18.50	−22.60	−26.00

The ANOVA test showed a highly significant difference ( *p* = 0.000) in Δa values at the different time intervals between the combined groups.

Coffee caused a significant variation in chromaticity from green to red after 24 hours of immersion ( *p* = 0.000). Lemonade showed similar behavior to artificial saliva for up to 2 weeks. Coca-Cola 0% showed similar behavior to artificial saliva for up to 4 weeks, respectively. Black tea significantly changed chromaticity from green to red at 1 week ( *p* = 0.020) and green tea significantly changed chromaticity from green to red at 2 weeks ( *p* = 0.000).

The ANOVA test showed a highly significant difference ( *p* = 0.000) in Δb values at the different time intervals between the combined groups. The two-by-two comparison showed that lemonade, coffee, and black tea significantly varied the chromaticity from blue to yellow after 24 hours. At 1 week, the chromaticity from blue to yellow changed for green tea compared with artificial saliva ( *p* = 0.000). At 2 weeks, Coca-Cola 0% showed a significant change in chromaticity from blue to yellow compared with artificial saliva ( *p* = 0.026); by the third week, the chromaticity from blue to yellow became similar to that of artificial saliva ( *p* = 0.260); after a month of immersion, the difference is again significant for Coca-Cola 0% ( *p* = 0.003).

The ANOVA test showed a highly significant difference ( *p* = 0.000) in ΔL values at the different time intervals between the combined groups. The Tukey's test revealed that the coffee showed a significant variation in lightness after 24 hours of immersion ( *p* = 0.000). Green tea significantly increased lightness after 1 week of immersion ( *p* = 0.005). This increase in lightness significantly exceeds that caused by black tea ( *p* = 0.001). Up to 3 weeks of immersion, black tea and Coca-Cola 0% did not significantly affect lightness.

### Color Measurement

Descriptive data relating to the variation of the color depending on the immersion solution are represented in Tables 10 and 11 . For Filtek Z350 XT, the ANOVA test showed a significant difference ( *p* = 0.000) in ΔE values at the different time intervals (between the combined groups). Tukey's test revealed that coffee, lemonade, and black tea caused a significant variation in color compared with the control group after 24 hours of immersion ( *p* = 0.000, 0.003, and 0.021, respectively). Coffee influenced the color more. Lemonade was equivalent to black tea ( *p* > 0.05). After 1 week of immersion, green tea significantly changed its color ( *p* = 0.000). Coca-Cola 0% showed similar behavior to artificial saliva throughout the experiment.

**Table 10:** Descriptive data of ΔE for the Filtek Z350 XT

		*N*	Mean value	Standard deviation	Minimum	Maximum
**Δ** E1 Filtek	Artificial saliva	5	2.39	1.704	1.12	5.39
	Coffee	5	17.93	3.69	13.11	21.50
	Lemonade	5	8.71	1.60	5.90	9.77
	Black tea	5	7.52	2.22	4.89	9.59
	Coca-Cola 0%	5	1.60	0.93	0.51	2.91
	Green tea	5	6.86	2.79	2.62	10.31
	Total	30	7.50	5.83	0.51	21.50
**Δ** E2 Filtek	Artificial saliva	5	2.83	1.19	0.87	3.95
	Coffee	5	24.82	4.52	18.72	29.97
	Lemonade	5	21.90	2.92	18.21	25.97
	Black tea	5	15.95	2.29	13.60	19.32
	Coca-Cola 0%	5	2.31	1.44	0.64	3.54
	Green tea	5	11.57	2.53	7.88	14.65
	Total	30	13.23	9.12	0.64	29.97
**Δ** E3 Filtek	Artificial saliva	5	2.17	1.01	1.18	3.30
	Coffee	5	25.23	3.25	21.44	29.31
	Lemonade	5	27.00	2.87	22.54	29.59
	Black tea	5	25.61	2.05	22.87	28.41
	Coca-Cola 0%	5	1.89	1.20	0.71	3.83
	Green tea	5	13.24	2.30	9.46	15.13
	Total	30	15.86	11.14	0.71	29.59
**Δ** E4 Filtek	Artificial saliva	5	2.08	0.972	1.15	3.24
	Coffee	5	29.66	2.66	26.52	32.90
	Lemonade	5	28.09	1.782	25.40	30.18
	Black tea	5	28.56	4.17	22.17	33.11
	Coca-Cola 0%	5	3.87	0.83	3.04	5.28
	Green tea	5	12.38	1.95	9.56	14.40
	Total	30	17.44	12.16	1.15	33.11
**Δ** E5 Filtek	Artificial saliva	5	3.69	1.40	1.46	5.30
	Coffee	5	28.21	3.62	23.08	31.80
	Lemonade	5	36.19	2.12	33.32	38.77
	Black tea	5	28.16	4.97	20.15	33.11
	Coca–Cola 0%	5	2.84	1.28	1.01	4.59
	Green tea	5	12.44	2.02	9.56	14.40
	Total	30	18.59	13.40	1.01	38.77

**Table 11:** Descriptive data of ΔE for the Harvard Restore

		*N*	Mean value	Standard deviation	Minimum	Maximum
**Δ** E1 Harvard	Artificial saliva	5	2.00	0.67	1.13	3.04
	Coffee	5	8.51	2.42	5.04	11.07
	Lemonade	5	2.69	0.78	1.92	3.82
	Black tea	5	3.70	2.38	0.87	7.27
	Coca-Cola 0%	5	1.43	0.67	0.85	2.55
	Green tea	5	5.13	2.81	1.28	8.91
	Total	30	3.91	2.96	0.85	11.07
**Δ** E2 Harvard	Artificial saliva	5	1.92	0.72	1.33	3.14
	Coffee	5	13.04	1.35	11.00	14.73
	Lemonade	5	5.01	2.91	1.81	9.31
	Black tea	5	12.36	2.07	9.02	14.16
	Coca-Cola 0%	5	2.43	0.59	1.95	3.24
	Green tea	5	15.8	3.88	12.29	21.10
	Total	30	8.43	5.95	1.33	21.10
**Δ** E3 Harvard	Artificial saliva	5	2.30	0.63	1.62	3.23
	Coffee	5	14.74	1.34	12.76	16.40
	Lemonade	5	5.32	2.30	2.62	8.87
	Black tea	5	19.39	1. 87	17.28	21.36
	Coca-Cola 0%	5	1.97	0.69	0.75	2.44
	Green tea	5	22.28	1.47	20.70	24.55
	Total	30	11.05	8.42	0.75	24.55
**Δ** E4 Harvard	Artificial saliva	5	2.47	1.15	1.15	4.19
	Coffee	5	21.85	1.97	19.25	23.33
	Lemonade	5	5.63	2.46	3.06	9.62
	Black tea	5	19.91	1.31	18.29	21.29
	Coca-Cola 0%	5	2.52	1.02	1.35	3.99
	Green tea	5	29.69	3.97	25.47	35.32
	Total	30	13.68	10.99	1.15	35.32
**Δ** E5 Harvard	Artificial saliva	5	1.96	1.23	0.51	3.27
	Coffee	5	21.68	1.70	19.77	24.36
	Lemonade	5	6.89	1.73	4.58	8.99
	Black tea	5	19.66	2.52	16.33	22.66
	Coca-Cola 0%	5	3.25	1.67	0.95	5.47
	Green tea	5	30.83	2.88	27.99	35.32
	Total	30	14.04	11.02	0.51	35.32

As for Harvard Restore, the ANOVA test showed a significant difference ( *p* = 0.000) in ΔE values at the different time intervals between the combined groups. The Tukey's test revealed that at 24 hours, only coffee significantly changed the color of Harvard ( *p* = 0.000). Black tea and green tea significantly changed the color of Harvard after 1 week of immersion ( *p* = 0.000 and 0.000, respectively)). The effect of the lemonade appeared between 2 and 3 weeks of immersion ( *p* = 0.043). Coca-Cola 0% showed similar behavior to artificial saliva throughout the experiment.

### Effect of Immersion on Color Difference

#### Green Tea

A nonsignificant difference was found between the two materials at 24 hours and 1 week ( *p* = 0.359 and 0.075, respectively). From 1 week to two weeks interval, the difference was significant ( *p* = 0.000) with the Harvard Restore which had the most modification.

#### Coca-Cola 0%

A nonsignificant difference was found between the two materials at day 0 to 24 hours ( *p* = 0.742), 24 hours to 1 week ( *p* = 0.864), 1 week to 2 weeks ( *p* = 0.898), 2 weeks to 3 weeks ( *p* = 0.052), and 3 weeks to 4 weeks ( *p* = 0.677).

#### Black Tea

A significant difference was found between the two materials at day 0 to 24 hours ( *p* = 0.031), 24 hours to 1 week ( *p* = 0.032), 1 week to 2 weeks ( *p* = 0.001), 2 weeks to 3 weeks ( *p* = 0.002), and 3 weeks to 4 weeks ( *p* = 0.009) with the Filtek Z350 XT having the most color change.

#### Lemonade

A significant difference between the two materials was revealed at different time intervals ( *p* = 0.000) with Filtek Z350 XT having the most color change.

#### Coffee

A very significant difference between the two materials was found at day 0 to 24 hours ( *p* = 0.001), 24 hours to 1 week ( *p* = 0.001), 1 week to 2 weeks ( *p* = 0.000), 2 weeks to 3 weeks ( *p* = 0.001), and 3 weeks to 4 weeks ( *p* = 0.007) with the Filtek Z350 XT having the most color change.

## Discussion

Our first null hypothesis was rejected. Our study showed that both composite resins tested showed discoloration after immersion in all beverages throughout the variable immersion period. As the immersion time increased, the color changes became more and more intense in accordance with the results of previous studies. 13 14

The highest discoloration occurred for 1 day to 7 days and when extended to 14 days, tended toward saturation. 15 16 Several studies have reported that the values of color difference (ΔE*) ranging from 1 to 3 are noticeable to the naked eye. The CIE Lab system has the advantage of being repeatable, sensitive, objective, universally accepted, and can measure small color changes.

Any color change (ΔE*) ranging from 3.3 to 3.7 and above is considered clinically noticeable. 17 In the present study, ΔE* ≥ 3.3 was taken as a noticeable and therefore clinically unacceptable color change.

In this study, all composite resins tested had color changes after a 7-day immersion in a coffee solution.

According to the previous studies, several factors may influence the degree of staining of composites such as incomplete polymerization, water absorption, 18 chemical reactivity, diet, oral hygiene, 19 20 and the surface condition of the restoration. 21 For this reason, all composite resin samples were light-cured on both sides for 40 seconds against two glass plates.

The solutions chosen were as follows: artificial saliva as a control, Coca-Cola 0% and lemonade as acidic solutions, and black tea, green tea, and Turkish coffee with a pH close to neutrality. These solutions are used quite frequently. 22

An immersion period of 28 days was chosen in accordance with two studies from 2010 and 2011 showing that the maximum immersion time used was 30 days. 13 23

Drinking a cup of coffee or tea takes an average of 15 minutes and, among coffee and tea drinkers, the average consumption is three cups per day. Therefore, a 28-day storage period simulates the consumption of these two beverages over 2 years, which is clinically relevant. 21 This is consistent with the study by Ertaş et al 15 who considered 28 days to be equivalent to approximately 2.5 years of aging (24 hours of *in vitro* staining corresponds to 1 month *in vivo* ). A longer immersion period may be required *in vivo* to induce clinically noticeable color changes. 17

This can be explained by the chewing and cleaning power of saliva on the one hand, and the daily brushing and rinsing to maintain good oral hygiene, on the other hand. 24 25

Garcia et al reported that the color of the composite resin changed over time, regardless of the immersion medium (saliva, Coca-Cola, tea). 26 Prodan et al 27 reported that specimens immersed in saliva showed color changes from their initial color and this was assumed to be due to the water absorption characteristics of the materials.

Our second null hypothesis postulating that the two materials tested have similar colorimetric behaviors was rejected. A review of the literature on this topic showed no work on Harvard Restore.

The value of ΔE* represents the relative value of color changes that an observer could report for the materials after or between immersion periods. Thus, ΔE* is more significant than the individual values of L*, a*, and b*. 28

Composite resins that can absorb water are also capable of absorbing other fluids with pigments, which then results in discoloration with the water acting as a vehicle for stain penetration into the resin matrix. 12

The composition of a composite resin and the relative amounts of organic matrix and filler content have an important influence on the colorimetric behavior of this material. Composite resins with a lower filler content and a higher amount of organic matrix tend to absorb more water at the matrix–filler interface, 14 leading to hydrolytic degradation of the inorganic phase. 17 Composite resins with higher filler content showed greater color stability. Indeed, the more numerous and smaller the fillers, the better the compactness and the less access to the organic matrix. The difference in performances of the two composites supports the effect of material composition as an intrinsic factor playing a role in their color change. Filtek Z350 XT (with a loading rate of e 63.3.5% by volume and a particle size range of 4–11 nm and containing bisphenol A glycidyl dimethacrylate [Bis-GMA], urethane dimethacrylate [UDMA]/ethoxylated bisphenol A dimethacrylate/triethylene glycol dimethacrylate [TEGDMA]) showed more discoloration than Harvard Restore (having 65% by volume and an average particle size of 0.05 to 1.5 µm and which contains mainly Bis-GMA). The differences could be related to the organic phase of the composite or to the filler volume or size, or to the porosity of the aggregated filler particles. 29 The degree of conversion has been mentioned as an important factor on the discoloration of composite resins. It is important that the composite resin has a uniform distribution of filler particles in the polymer matrix thus minimizing the formation of filler-rich and filler-depleted zones in the composites. This is particularly important with respect to the performance of composites in aqueous environments, as voids or unbound spaces at the filler–matrix interface can increase water absorption. 30

Finishing and polishing procedures influence the surface quality and can therefore be related to the resistance of resin-based materials to staining agents. 31

The colorations observed in the studies are generally those that occur on the surface. This is due to the hydrophobic monomers in the composition of the composite resins. For example, UDMA-based monomers show lower color variation values than other types of dimethacrylate-based monomers. This can be explained by the low viscosity and low water absorption of urethane dimethacrylate as well as its polymerization with visible light. 28

Incorporation of higher amounts of TEGDMA results in increased water uptake in Bis-GMA-based resins. 28 Imazato et al 32 reported that this was due to increased surface hydrophilicity. Hydrophilic groups such as the ethoxy group in TEGDMA had an affinity for the water molecule through hydrogen bonding to oxygen. 33 Composites that have TEGDMA in their composition release a large number of monomers into aqueous media compared with those based on Bis-GMA and UDMA, resulting in greater color fading. 34 In several studies, greater discoloration was obtained in composite resins that include TEGDMA, which could be responsible for the high degrees of water absorption and discoloration. 34 In our study, Filtek Z350 XT contains both TEGDMA and UDMA, while Harvard Restore contains only Bis-GMA, which explains its better colorimetric behavior.

The results obtained in the present study showed that all samples experienced color changes from the initial measurements. For the Filtek Z350 XT, the greatest color change was caused by coffee followed by lemonade and black tea followed by green tea, Coca-Cola 0%, and artificial saliva. For the Harvard Restore, the greatest color change in all groups was caused by green tea followed by coffee, black tea, lemonade, Coca-Cola 0%, and artificial saliva. Thus, our third null hypothesis was rejected.

In this study, coffee was used as a staining agent because of its frequent consumption in daily life. This beverage can cause brown and yellow stains to appear on teeth as well as on the surfaces of composite resin restorations. 28

For coffee, there was a change in the color of the resin from the first 24 hours of immersion, this is consistent with the observations of Domingos et al 35 and Yazici et al. 14

The staining ability of coffee on composites can be justified by the staining sensitivity of composite resins attributed to their degree of water absorption and the hydrophilicity of the resin matrix. 12

Coffee can cause color changes both by adsorption and absorption of its dyes onto/in the organic phase of composite resins. 28

Coffee has been found to have a stronger chromatogenic character than tea or Coca-Cola. The yellow dyes in coffee are less polar than in tea. The absorption and penetration of the dyes into the organic phase of the resins is probably due to the compatibility of the polymer phase with the yellow coffee dyes. In addition, tea and coffee contain a large amount of coloring agents such as gallic acid, which could be another reason for the coloring ability of these solutions. 36 This idea is in agreement with the study conducted by Nordbö et al. 37

Our results showing that the discoloration potential of coffee is higher than that of tea or Coca-Cola 0% are in line with the studies conducted by Topcu et al 28 and Domingos et al. 35 Previous studies have shown that the addition of sugar and milk powder in tea and coffee leads to a greater change in color. The low pH may affect the surface integrity by softening the organic matrix, causing loss of structural ions and affecting the wear resistance of dental materials. However, in the study by Catelan et al, 23 the low pH of cola (2.36) did not influence the color change, as higher pH solutions such as orange juice (3.39) and red wine (3.41) cause greater staining. The caramel color in the Coca-Cola solution causes color changes from the palest yellow to the deepest brown. 36 However, according to Bagheri et al 29 and Ertaş et al, 15 the lack of yellow dye in Coca-Cola resulted in less discoloration than coffee and tea. In our study, the samples immersed in Coca-Cola 0% showed a slight color change, which could be due to the change in surface roughness of the samples owing to the low pH (2.84) of the solution. This further contributes to the adsorption of the color on its surface. These results are in agreement with Patel et al 38 who stated that Coca-Cola causes minimal color change in composite resins. Although Coca-Cola has the lowest pH which could damage the surface integrity of the materials, it did not produce as much discoloration as coffee. In our study, the color variation between lemonade and Coca-Cola 0% was significant from the first few immersion intervals. Indeed, Coca-Cola soft drink contains carbonic acid 39 and phosphoric acid and lemonade contains citric acid. Acids behave differently in promoting dissolution and thus erosion of materials. In addition, the presence of phosphate ions in Coca-Cola may suppress dissolution because it has been shown that these ions can reduce the rate of dissolution of calcium phosphate from the tooth. 2 Thus, the different acids present in the beverages could explain these results.

According to Nasim et al, the staining ability of tea could be due to the presence of tannic acid. 13

When comparing the effects of different beverages, coffee generally produced the worst discolorations, followed by lemonade, black tea, green tea, and Coca-Cola. These beverages had negative ΔL* and positive Δb* values, indicating that the materials became darker and more yellow, respectively. These three drinks had varying effects on Δa*. This could be due to the variation in the dyes present in the three drinks.

Although acidity is measured as pH, the total amount of acid present (titratable acidity) may be a better indicator. This is determined by titration against a standard sodium hydroxide solution. The correlation between pH and titratable acidity is not apparent, which means that a beverage can have both a high pH and titratable acidity. Coffee and tea had a large number of acids present in their composition and may have had a significant total acidity. This could have increased the chemical erosion of material surfaces and the materials and aggravated discoloration. 22

Our study is an *in vitro* study, allowing staining of the material on both sides. In a clinical situation, the material is bonded to a tooth structure and is exposed to solutions and light on only one side. 40 The specimens had flat surfaces, whereas clinically, composite resin restorations have an irregular shape with convex and concave surfaces.

The results of this study need to be corroborated by clinical studies. Further clinical and *in vitro* studies are needed to evaluate the color behavior of the microhybrid composite resin against other beverages and nutrients.

## Conclusion

Within the limitations of this current study, it was concluded that:

- Harvard Restore showed high resistance to fading compared with Filtek Z350.
- Coffee and black tea had the strongest effects on the discoloration of the composites. especially for Filtek Z350.
- Coca-Cola showed similar behavior to artificial saliva. Both solutions caused minimal discoloration of both composite resins.
- The duration of the immersion influenced the intensity of the discoloration.
